# Is the Anamnesis Enough to De-Label Patients with Reported Beta-Lactam Allergy?

**DOI:** 10.3390/jcm13237267

**Published:** 2024-11-29

**Authors:** Lesia Rozłucka, Barbara Rymarczyk, Radosław Gawlik, Joanna Glück

**Affiliations:** Department of Internal Medicine, Allergology and Clinical Immunology, Faculty of Medical Sciences in Zabrze, Medical University of Silesia, 40-752 Katowice, Poland

**Keywords:** beta-lactam, hypersensitivity, de-labeling, risk stratification

## Abstract

**Background:** The decision whether to de-label patient with suspected BL hypersensitivity is based on risk stratification. The aim of this study was to prepare a characteristic of diagnostic risk groups and to create a model enabling the identification of the low-risk diagnostic group. **Methods:** We analyzed the medical records of patients hospitalized due to suspected hypersensitivity to BL antibiotics. Based on their medical-history data, patients were divided into three diagnostic risk groups, using the criteria proposed by Shenoy et al. Univariate and multivariate analysis models were used to create a diagnostic tool. **Results:** Among 263 patients referred for BL hypersensitivity diagnosis, 88 (33.5%) were allocated to group I, 129 (49%) to group II, and 46 (17.5%) to group III. There were significant differences between diagnostic risk groups regarding history of hypersensitivity to penicillins (*p* < 0.001), cephalosporins (*p* < 0.001), >1 BL (*p* < 0.05), several episodes of BL hypersensitivity (*p* < 0.001), medical intervention (*p* < 0.001), documented hypersensitivity (*p* < 0.001), time from drug intake to symptoms (*p* < 0.001), and time from hypersensitivity to diagnosis (*p* < 0.001). In total, 81 patients (30.8%) were de-labeled: 52 (59.8%) in group I, 27 (20.9%) in group II, and 2 (4.3%) in group III. The univariate analysis model of the low-diagnostic-risk group applied to the de-labeled part showed 90% specificity and 21.93% sensitivity. NPV and PPV were estimated at 72.04% and 49.53%, respectively. The multivariate model had high specificity but low sensitivity; its NPV was 76%, with 68% PPV. **Conclusions:** The tool enabling the identification of low-diagnostic-risk patients based on anamnesis is not sensitive enough to de-label patients on its basis.

## 1. Introduction

Beta-lactam (BL) antibiotics are one of the most common causes of drug hypersensitivity [1,2,3]. Approximately 6–10% of the population reports an “allergy” to penicillin (PN), including 15% of hospitalized patients, but allergy workup excludes hypersensitivity in about 95% of them [4,5,6,7,8]. Actual hypersensitivity to cephalosporins (CF) is estimated at <2% [2] and to carbapenems is 0.3–3.7% [9], while actual hypersensitivity to monobactams is unknown. Hypersensitivity in the BL group may involve a BL ring, a single BL antibiotic, or antibiotics with a similar or identical structure of R1 side chain, less often R2 [10,11,12,13]. Anaphylaxis after BL administration is rare, with a frequency of 46.9 per 10,000 for penicillins and 6.1 per 10,000 for cephalosporins in the general population. At the same time, penicillins are the most common cause of drug-induced anaphylaxis [14].

The diagnosis of “allergy” to BL antibiotics without verification, is associated with a longer hospital stay; more frequent use of fluoroquinolones, vancomycin, and clindamycin; and higher rates of *Clostridium difficile*, methicillin-resistant *Staphylococcus aureus* (MRSA) and vancomycin-resistant enterococci (VRE) infections compared to patients without the “allergy” label [15,16]. “Allergy” to BL antibiotics history is associated not only with negative consequences for the patient, but also for the healthcare system by increasing the cost of treatment [17,18,19]. Avoidance of BL antibiotics in patients reporting an “allergy” results in a higher risk of treatment failure [20].

De-labeling involves removing the “allergy” label reported by the patient. There are several de-labeling strategies:

- Based only on the medical history, if anamnesis clearly indicates symptoms other than hypersensitivity (e.g., isolated, uncharacteristic gastrointestinal symptoms, headaches, and hypersensitivity to BL antibiotics in the family);
- Diagnosis based on skin tests with determinants, followed by provocation with a single dose of the drug or in divided doses;
- Direct oral drug provocation without prior skin testing [21,22].

The choice of de-labeling method is based on the risk stratification. The main goal of risk stratification is to select a group of high-risk patients who require access to specialized diagnostics first. At the same time, due to the rare actual hypersensitivity reactions to BL antibiotics, it is important to select a low-risk group in which de-labeling can be safely performed. Previous European Academy of Allergy and Clinical Immunology (EAACI) recommendations suggested a full allergy workup in patients with suspected BL antibiotic hypersensitivity, without assessing the severity of the reaction [23,24,25]. The current EAACI position paper recommends risk stratification, assessing time and severity of the hypersensitivity reaction. Despite the proposed high- and low-risk groups, diagnosis without skin testing is recommended only in a narrow group of patients, including pediatric patients with benign maculopapular exanthema (MPE) and patients with palmar exfoliative exanthema [26]. In the last decade, many papers on risk stratification in BL hypersensitivity were published regarding mainly penicillins [27,28,29,30,31]. In contrast, recent United States guidelines suggested several important changes in the recommendations for BL hypersensitivity diagnosis, including direct de-labeling patients with nonspecific symptoms of penicillin hypersensitivity and direct provocation with CF with a different R1 side chain in patients with non-anaphylactic hypersensitivity to CF and anaphylactic hypersensitivity to PN. In patients with a history of unverified non-anaphylactic PN/CF allergy, the use of CF/PN without prior skin testing is suggested [4]. Both in the United States and Europe, the number of patients reporting hypersensitivity to BL antibiotics (mainly penicillins) far exceeds the number of specialists who could verify the “allergy” label [32]. The purpose of this study was to analyze the group of patients reporting hypersensitivity to BL antibiotics and to create a model identifying low-risk patients based on anamnesis.

## 2. Materials and Methods

### 2.1. Study Design

We conducted a retrospective, single-center, observational study based on medical records analysis. We analyzed medical records of patients hospitalized at the Department of Allergology and Clinical Immunology between January 2018 and June 2022 due to suspected hypersensitivity to BL antibiotics. Among patients with reported hypersensitivity to various drugs, we selected a group of patients referred for diagnosis of antibiotics hypersensitivity. Then we excluded patients with suspected hypersensitivity to antibiotics other than BL. The final analysis included only patients reporting hypersensitivity to at least one BL antibiotic.

Based on the history data, patients were divided into three diagnostic risk groups, using the criteria proposed by Shenoy et al. [33]. The low-risk group (group I) included isolated reactions that are unlikely allergic (e.g., gastrointestinal symptoms and headaches), pruritus without rash, remote (>10 y) unknown reactions without features of IgE, and family history of penicillin allergy. The moderate-risk group (group II) included urticaria or other pruritic rashes, and reactions with features of IgE but not anaphylaxis. The high-risk group (group III) included anaphylactic symptoms, positive skin testing, recurrent reactions, and reactions to multiple β-lactam antibiotics. The criteria were applied to patients reporting hypersensitivity either to penicillins or to cephalosporins, or to both. For the purpose of the study, hypersensitivity to cephalosporins was divided into three groups according to the R1 side-chain structure based on Romano et al. [34].

### 2.2. Ethics

The study was approved by the Bioethics Committee at the Medical University of Silesia in Katowice (PCN/0022/KB1/123/19) and was in accordance with the ethical principles for human experimentation initiated by the Declaration of Helsinki. The necessity for written informed consent from the patients was removed because of the retrospective nature of the study. The investigation was carried out using an anonymized dataset; therefore, patient confidentiality was preserved.

### 2.3. Statistical Analysis

For quantitative traits, due to the non-normal distribution in at least one group, the non-parametric *Kruskall–Wallis* (KW) test was used. For qualitative variables, statistical analysis was performed using a non-parametric test of independence x^2^. Linear model with a logit binding function was used to evaluate the model for membership in the low-risk group (group I). Results are presented as odds ratio with 95% confidence interval. Multivariate modeling was performed using a stepwise backward method, manually including age and gender as potential clinically relevant confounding variables in the model. Calculations were performed using the R language in the RStudio software (PBC, Boston, MA, USA. http://www.posit.co/). A *p* < 0.05 was considered statistically significant.

## 3. Results

Between January 2018 and June 2022, there were 1957 patients referred to the department due to the drug hypersensitivity diagnosis. Among them, 391 patients were hospitalized due to antibiotic hypersensitivity diagnosis. From this group, we excluded 128 patients hospitalized due to the diagnosis of hypersensitivity to antibiotics other than BL. Our final analysis included 263 patients classified into three groups. The low-risk group consisted of 88 (33.46%) patients, moderate risk of 129 (49.05%) patients (group II), and the remaining 46 (17.49%) patients were classified into the high-diagnostic-risk group (group III).

### 3.1. General Characteristic of the Diagnostic Risk Groups

Gender, the prevalence of seasonal and perennial rhinitis, asthma, and smoking did not differ significantly between the diagnostic risk groups. Detailed data are shown in Table 1.

### 3.2. Anamnestic Data

The frequency of reported hypersensitivity incidents to penicillins and to aminopenicillins significantly decreased as the diagnostic risk increased, (*p* < 0.001). Reported hypersensitivity to all cephalosporins and cephalosporins with methoxyimino group in the R1 side chain (group A) was significantly more common, along with increasing diagnostic risk (*p* < 0.001). There were no significant differences between groups in the frequency of reporting hypersensitivity to non-BL antibiotics and other drugs. In the low-risk group (group I), patients were significantly more likely not to remember the time between taking the drugs and the onset of hypersensitivity symptoms (*p* < 0.01) and to report several episodes of hypersensitivity to the BL antibiotic (*p* < 0.01), compared to the moderate- and high-risk groups. Short time from BL ingestion to the reported onset of hypersensitivity symptoms, the need for medical intervention, and documented anaphylaxis occurred significantly more often in the high-risk group (group III). Due to too small numbers in the groups, the calculation of statistical significance was not possible in all the data. Detailed characteristics are shown in Table 2.

### 3.3. De-Labeling in the Risk Groups

After a full diagnostic workup, including skin tests and drug provocation, de-labeling was performed in 81 patients (30.8%): 52 patients (59.8%) in group I, 27 patients (20.9%) in group II, and 2 patients (4.3%) in group III (*p* < 0.001).

### 3.4. Analysis of a Low-Risk Group Hypersensitivity to Beta-Lactam Antibiotics

#### 3.4.1. Univariate Analysis of a Low-Risk Group

An increased chance of being assigned to a low-risk group (group I) was observed among patients who declared hypersensitivity to more than one BL antibiotic. The chance of being assigned to group I increased threefold in patients who reported hypersensitivity to penicillins upon admission. A declared time from BL antibiotic ingestion to symptom onset of less than 1 h strongly decreased the chance of being in the group I, while time longer than 24 h increased the chance of being assigned to group I. The chance of being assigned to group I was increased by declaring an unknown time to symptoms and indicating the occurrence of more than one hypersensitivity episode. Patients diagnosed several years apart from the hypersensitivity episode were less likely to be assigned to group I, while those whose time to reaction was several decades or unknown were assigned to group I twice as often. Detailed data are shown in Table 3. The model applied to the de-labeled part of the low-risk group in comparison to the rest of the group showed 90% specificity and 21.93% sensitivity. NPV and PPV were estimated at 72.04% and 49.53%, respectively.

#### 3.4.2. Multivariate Analysis of a Low-Risk Diagnostic Group

**Figure 1:**
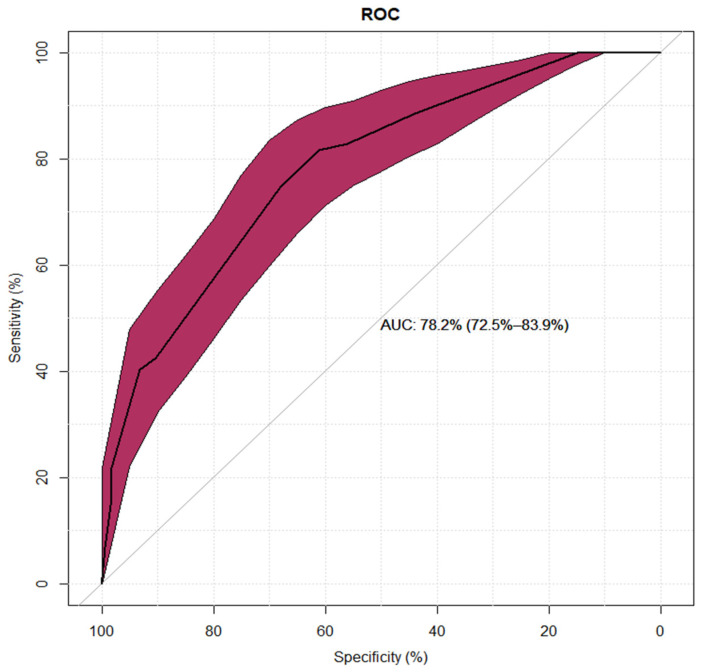
Sensitivity and specificity for the multivariate logistic regression model of the low-diagnostic-risk group shown as a ROC (Receiver Operating Characteristic) curve.

In a multivariate model, diagnostic time of several years since reported hypersensitivity to diagnosis significantly reduced chance of belonging to group I. More than one episode of hypersensitivity to BL more often characterized patients with low diagnostic risk, as did prolonged drug reaction time (>24 h). A short reaction time occurs more often in the moderate- and high-risk groups. Detailed results are shown in Table 4. The model had high specificity but low sensitivity; its negative predictive value was 76%, with a 68% positive predictive value (Figure 1).

## 4. Discussion

In our study, we characterized patients with low, moderate, and high risk of diagnosis of hypersensitivity to BL antibiotics based on the medical history data. The groups of low, moderate, and high diagnostic risk of hypersensitivity to BL antibiotics differ in regard to their history data, such as frequency of reporting hypersensitivity to penicillins, cephalosporins, and to more than one BL antibiotic; occurrence of several episodes of hypersensitivity to BL antibiotics; need for medical intervention, documented anaphylactic reaction; prolonged time from drug intake to reaction; and prolonged time from hypersensitivity reaction to diagnosis. We also attempted to create a diagnostic tool, that would identify a low-diagnostic-risk group based only on medical history data. The tool had a high specificity but, unfortunately, low sensitivity to carry out risk stratification decisions based on it.

Inglis et al. analyzed 5023 electronic databases with reported penicillin ADRs (adverse drug reaction). Among them, penicillin allergy was diagnosed in 4773 (95%) and intolerance in 250 (5%). The most reported drug was “penicillin”, “penicillins”, or “BL antibiotic” in 3992 patients (79.5%), and among them, amoxicillin was the most common, at 866 (17.2%). Among 4979 patients, about half of them—2549 (50.7%)—were classified as low risk; 1378 (27.4%) were classified as high risk; and in 1052 (20.9%) of them, the risk could not be assessed [35]. In a retrospective study by Albin et al., rash and unknown/undocumented hypersensitivity reaction to penicillins accounted for more than half (57.2%) of the hypersensitivity symptoms reported by patients; urticaria was reported in 18.9%, angioedema in 11.8%, and anaphylaxis in 6.8%. At the same time, among patients reporting anaphylaxis, 92.4% had no medical records confirming the reaction [36]. Vyles et al. analyzed a pediatric group of 500 patients. Among them, 380 (76%) children had symptoms of low-risk hypersensitivity to penicillins. The most reported symptoms were rash, at 466 (92.8%) patients, and pruritus, at 203 (40.6%) patients. Among 120 children with at least one high-risk symptom, the most common manifestation of hypersensitivity was angioedema—50 (10%) [37]. A similar study was conducted in a pediatric group admitted to the emergency department due to hypersensitivity to cephalosporins. In 75 of 128 patients (58.6%), only low-risk symptoms were described, and the most common symptoms were skin rash and pruritus (73%) appearing at least 6 h after taking the drug. In the high-risk group, the most common symptom was also a rash, but appearing within 6 h of BL antibiotic administration [38].

A study by Touati et al. conducted between 1992 and 2018 at the Department of Allergology at the Montpellier Clinical Hospital included 476 patients who reported DHR (drug hypersensitivity reaction) after cephalosporin use. Among them, 83 patients reported anaphylactic shock; 85 reported anaphylaxis; and 308 reported “other” reactions, which included urticaria, angioedema, MPE, and severe cutaneous adverse reaction (SCAR) [39]. Anaphylaxis after cephalosporin administration is rare. In an analysis by Macy et al., anaphylaxis was reported in 5 out of 901,908 oral administrations of cephalosporins and in 8 out of 487,630 parenteral administrations of cephalosporins [40].

In our study, as in the study cited above, patients reporting hypersensitivity to penicillins, mainly aminopenicillins, mostly belonged to the low-risk group, as well as patients who could not identify the specific “penicillin” causing the hypersensitivity. Over the past few decades, the use of parenteral penicillin has significantly decreased; therefore, the incidence of severe hypersensitivity, including anaphylaxis, is rare [41]. Both our findings and those quoted above may also be due to the fact that most reactions after penicillins are mild skin rashes, such as MPE, which is not a life-threatening condition for the patient; therefore, such patients belong to the low-diagnostic-risk group. On the contrary to the studies cited above, patients with a history of hypersensitivity to cephalosporins, including group A cephalosporins, were significantly more likely to be in the high-risk group. In our study, data regarding group A are similar with the results of a multicenter, retrospective cohort study by Yang et al. In their study, group I of cephalosporins (ceftriaxone, cefuroxime, cefepime, cefotaxime, and ceftizoxime) were the most common cause of anaphylaxis induced by cephalosporin administration [42]. In our study, patients reporting hypersensitivity to group C cephalosporins, mainly cefazolin, were mostly in the high-risk group. This may be due to the fact that cefazolin is administered prophylactically in the perioperative period intravenously, resulting in more severe hypersensitivity reactions. Similar observations were made by Bogas et al. in a study that examined a group of 184 patients, among whom 76 (41.3%) had confirmed hypersensitivity. Hypersensitivity was confirmed in all patients reporting anaphylactic shock and the majority reporting anaphylaxis, while in the group with mild reactions, all of the patients reporting skin rush and pruritus were de-labeled [43].

In our analysis, patients who reported hypersensitivity to more than one BL antibiotic were significantly more often assigned to the low-risk group. This may be due to the simultaneous use of several antibiotics by the patient during infection and the inability to determine which drug caused the reaction. This may be similar regarding non-BL antibiotics and other drugs (most often NSAIDs), although our analysis did not find significant differences between studied groups. Hypersensitivity to drugs from unrelated groups is a rare phenomenon [44,45].

A systematic review by Jagpal et al. revealed inaccuracies in the nomenclature and diagnosis of multiple drug intolerance syndrome (MDIS), multiple drug allergy syndrome (MDAS), and multiple drug hypersensitivity syndrome (MDHS) [44]. Blumenthal et al. described MDAS as a hypersensitivity reaction, with a probable immune mechanism involved, to at least two drugs from different, unrelated groups. The incidence of MDAS was estimated at about 1%, while MDIS was estimated at about 6%. In contrast, Voelker et al. identified multidrug hypersensitivity as a risk factor for IgE-mediated penicillin drug allergy in a pediatric population (OR = 2.19, 95% CI 1.3–3.6, *p* = 0.0019) [46]. The diagnosis of multidrug hypersensitivity, without verification, can be fatal for the patients because, in a situation requiring immediate intervention, those patients may be left without antimicrobial treatment options.

In our study, patients reporting several episodes of hypersensitivity to BL were significantly more often assigned to the low-diagnostic-risk group. In the high-diagnostic-risk group, only 15% reported more than one reaction after administration of BL antibiotic. Probably due to the fear of retaking a drug that previously caused a hypersensitivity reaction and an unequivocal relationship between the drug and hypersensitivity symptoms, usually with a severe course. Study by Park et al. concluded that previous administration of any BL antibiotic significantly increased the risk of anaphylaxis in patients reporting hypersensitivity to cephalosporins (*p* = 0.01), but not to penicillins (*p* = 0.23). Interestingly, in multivariate regression, there was no statistical significance between re-exposure to BL antibiotics and increased risk of BL-induced anaphylaxis [47].

### Characteristics of the Low-Diagnostic-Risk Group

A diagnostic tool based on anamnesis could help to identify low-risk patients who could be safely de-labeled. A complete workup, including skin and provocation tests, would not be necessary in the abovementioned group. This would reduce the wait time for diagnosis in priority to high-risk patients.

Australian researchers presented a simple tool “PEN-FAST”, which has been validated both internally and by researchers from the United States and Europe [30,48]. In a multivariate analysis involving 622 subjects, four factors associated with penicillin hypersensitivity were summarized. They created the mnemonic “PEN-FAST” in which “PEN” indicates penicillin hypersensitivity (2 points), “F” is the time since the reaction within five years (2 points), “A” is anaphylaxis/angioedema (2 points), “S” is SCAR (2 points), and “T” is the need for therapeutic intervention (1 point). Respondents with less than three points were classified as low-risk patients. In internal validation, the tool’s sensitivity was estimated at 71%, with a specificity of 79%, PPV of 25%, and NPV of 96% [30]. External validation in a group of 995 subjects from three centers showed a sensitivity of 70.4–87.5% and an NPV of 84.9–98.4% [49].

We attempted to create a model based on anamnesis alone that would characterize low-risk patients using a multivariate logistic regression. In the univariate model, penicillin hypersensitivity, time from drug intake to symptoms >24 h/unknown, long duration or unknown time from a hypersensitivity episode to diagnosis, and more than one episode of hypersensitivity to BL antibiotics were significant predictors of being assigned to the low-diagnostic-risk group. Regarding the de-labeled group, the specificity of the tool was estimated at 90%, with a sensitivity of 21.93%, NPV of 72.04%, and PPV of 49.53%. In the multivariate model, the time from drug intake to symptom onset of more than 24 h and more than one episode of hypersensitivity to BL antibiotics increased the chance of being in the low-risk group by several times. The NPV was 76%, and the PPV was 68%. Contrary to our expectations, we found a low PPV in the univariate analysis and a slightly higher PPV in the multivariate analysis; this may be due to the fact that the tool was created using only subjective data reported by patients. It is likely that an increase in PPV could have been obtained by including objective interview data, e.g., based only on entries from medical records, or expanding the tool with results of skin tests and provocation tests. However, our aim was to create a tool that could be useful in risk stratification and de-labeling beta-lactam allergy by specialists other than allergologists.

Similar observations were described by Chiriac et al. in a study consisting of two parts, a retrospective analysis based on which a predictive model was created, and a prospective study in which the prepared model was validated. Using logistic regression, sensitivity for the retrospective and prospective models was estimated at 51% and 60%, respectively, with specificity at 75% and 80%, PPV at 40% and 57%, and NPV at 83% and 82%. The decision tree model presented high specificity, but it showed low sensitivity, i.e., <45%. Both methods were unable to accurately predict the occurrence of BL hypersensitivity to antibiotics [50].

In a multicenter Australian study, Stevenson et al. presented eight low-risk models. In the most precise model, which included isolated skin rash and a time from hypersensitivity reaction longer than one year, the sensitivity was 80.6%, specificity was 60.8%, and NPV was 94.7% [51].

Siew et al. identified a cohort of low-risk patients who could not remember the name of the BL antibiotic causing the hypersensitivity reaction, reported a history of no anaphylaxis, and had a hypersensitivity reaction at least one year before diagnosis. In this group, the medical history data had a comparable negative predictive value for the occurrence of type I hypersensitivity to BL antibiotics (98.4%), e.g., negative skin test results (98.9%) [52].

The algorithm based on anamnesis proposed by Soria et al. showed high sensitivity (92.7%) with low specificity (35.8%) and NPV of 96.3% [53].

Devchand et al. developed a validated tool that can be used by non-allergist physicians to make further diagnostic decisions based on risk stratification. The tool takes into account data from the patient’s history and laboratory test results [31,54,55].

In a recently published study, Dunham et al. presented a digital tool designed to simplify decision-making for physicians from other specialties in regard to patients with a history of BL antibiotic hypersensitivity. The tool encourages appropriate antibiotic selection for low- and moderate-risk groups, and to be extra cautious with high-risk groups [56].

Telemedicine is also usable in the risk stratification of BL antibiotic hypersensitivity. It does not completely replace diagnostics, but by identifying low-risk patients who can be safely de-labeled without an in-person visit, it can reduce waiting times to see a specialist and costs to the healthcare system [57,58].

The limitations of our analysis include its retrospective, single-center nature. Some complaints reported by patients, such as shortness of breath, are subjective and not verifiable; therefore, those patients may have been misclassified into particular risk groups. Expanding the study with the pediatric population would increase the value of this study. In conclusion, the data obtained may improve the knowledge on risk stratification in patients hospitalized due to suspected BL hypersensitivity. Further research is needed to identify risk factors for BL antibiotic hypersensitivity and to enable the development of more precise, easily accessible diagnostic tools for removing the label of BL allergy. This would result in both health benefits for the patient and financial benefits for the healthcare system. The diagnostic tool based only on anamnesis does not clearly identify low-risk patients, who could be safely de-labeled.

## 5. Conclusions

The tool enabling the identification of low-diagnostic-risk patients based on anamnesis is not sensitive enough to de-label patients on its basis.

## Figures and Tables

**Table 1:** General characteristics of patients in diagnostic risk groups.

General Characteristics	I n = 88 (33.46%)	II n = 129 (49.05%)	III n = 46 (17.49%)	*p*-Value
Gender (F)	76 (86.4)	106 (82.2)	34 (73.9)	ns
Age (years; mean ± SD)	49.5 ± 17.6	47.45 ± 15.6	45.8 ± 15.5	ns
SAR	12 (13.6)	20 (15.5)	6 (13)	ns
PAR	5 (5.7)	10 (7.8)	3 (6.5)	-
Asthma	10 (11.4)	11 (8.5)	5 (10.9)	ns
Smoking	15 (16.1)	24 (18)	10 (21.7)	ns

F—indicates female, ns—non-significant, PAR—perennial allergic rhinitis, SAR—seasonal allergic rhinitis.

**Table 2:** Data reported by the patients in the diagnostic-risk groups.

Reported Data	I n = 88 (33.46%)	II n = 129 (49.05%)	III n = 46 (17.49%)	*p*-Value -Test χ^2^
PN hypersensitivity (n = 198, 75.29%)	77 (87.5)	102 (79.1)	19 (41.3)	*p* < 0.001
Benzylpenicillin (n = 5)	3 (3.4)	2 (1.6)	0 (0)	-
Phenoxymethylpenicillin (n = 2)	1 (1.1)	0 (0)	1 (2.2)	-
Procaine penicillin (n = 2)	2 (2.3)	1 (0.8)	0 (0)	-
Aminopenicillin (n = 170)	62 (70.5)	90 (69.8)	18 (39.1)	*p* < 0.001
“Some” PN (n = 36)	15 (17)	18 (14)	3 (6.5)	-
CF hypersensitivity (n = 105, 39.92%)	28 (31.8)	47 (36.4)	30 (65.2)	*p* < 0.001
Group A CF (n = 83, 31.56%)	23 (26.1)	38 (29.5)	22 (47.8)	*p* < 0.001
Cefuroxime (n = 77)				
Ceftriaxone (n = 5)				
Cefixime (n = 1)				
Group B CF (12, 4.56%)	5 (5.7)	6 (4.7)	1 (2.2)	-
Cefadroxil (n = 5)				
Cefaclor (n = 4)				
Cephalexin (n = 3)				
Group C CF (n = 12, 4.56%)	1 (1.1)	4 (3.1)	7 (15.2)	-
Cefazoline (n = 11)				
Cefamandole (n = 1)				
“Some” CF (n = 3, 1.14%).	2 (2.3)	1 (0.8)	0 (0)	-
Hypersensitivity to >1 BL antibiotic (n = 62, 23.57%)	24 (27.3)	34 (26.3)	4 (8.7)	*p* < 0.05
Hypersensitivity to non-BL antibiotics (n = 80, 30.42%)	27 (30.7)	42 (32.6)	11 (23.9)	ns
Macrolides	11 (12.5)	22 (17.1)	6 (13)	ns
Quinolones	3 (3.4)	3 (2.3)	0 (0)	-
Aminoglycosides	3 (3.4)	5 (3.9)	2 (4.3)	-
Tetracyclines Glycopeptides	7 (8) 0 (0)	6 (4.7) 1 (0.8)	3 (6.5) 0 (0)	- -
Sulfonamides	9 (10.2)	11 (8.5)	4 (8.7)	ns
Lincosamides	4 (4.5)	5 (3.9)	1 (2.2)	-
Hypersensitivity to other drugs (n = 107, 40, 68%)	39 (44.3)	52 (40.3)	16 (34.8)	ns
NSAIDS	28 (31.8)	37 (28.7)	10 (21.7)	ns
LA	3 (3.4)	5 (3.9)	3 (6.5%)	-
General anesthesia	1 (1.1)	2 (1.6)	1 (2.2)	-
Contrast agents	3 (3.4)	1 (0.8)	2 (4.3)	-
Heparins	1 (1.1)	1 (0.8)	0 (0)	-
Time from BL antibiotic intake to onset of symptoms				
<1 h (n = 97, 36.88%).	11 (12.5)	46 (35.7)	40 (87)	*p* < 0.001
≥1–24 h (n = 50, 19.01%).	15 (17)	33(25.6)	2 (4.3)	-
>24 h (n = 29, 11.02%).	21 (23.9)	8 (6.2)	0 (0)	-
No data (n = 78, 29.66%)	36 (40.9)	38 (29.5)	4 (8.7)	*p* <0.001
>1 episode of hypersensitivity to BL antibiotic (n = 72, 27.38%)	33 (37.5)	32 (24.8)	7 (15.2)	*p* < 0.001
Medical intervention (n = 156, 59, 32%)	25 (28.7)	85 (66.4)	46 (100)	*p* < 0.001
Documented anaphylactic reaction (n = 76, 28.9%)	2 (2.3)	34 (26.4)	40 (87)	*p* < 0.001
Time from hypersensitivity episode to diagnosis				
6 wks–6 months. (n = 21, 7.98%)	4 (4.5)	10 (7.8)	7 (15.2)	ns
>6 months–year (n = 51, 19.39%)	12 (13.6)	20 (15.5)	19 (41.3)	*p* < 0.001
Several years (n = 83, 31.56%)	19 (21.6)	52 (40.3)	12 (26.1)	*p* < 0.05
10–20 years (n = 29, 11.03%)	13 (14.8)	13 (10.1)	3 (6.5)	-
Several decades (n = 29, 11.03%)	15 (17)	12 (9.3)	2 (4.3)	-
Unknown (n = 47, 17.87%)	23 (26.1)	22 (17.1)	2 (4.3)	-

BL—beta-lactam, CF—cephalosporins, LA—local anesthetics, non-BL—non-beta-lactam, ns—non-significant, NSAID—non-steroidal anti-inflammatory drugs, PNs—penicillins.

**Table 3:** Univariate logistic regression model of the low-diagnostic-risk group presented as odds ratio (OR) with 95% confidence interval.

	Single-Factor Model			
Variable	*p*	OR	95% CI	
Gender (F)	0.22	1.56	0.77	3.19
Age < 45 vs. ≥45 years old	0.26	1.01	0.99	1.03
BMI < 25 vs. ≥25	0.24	0.97	0.93	1.02
SAR, PAR	0.92	0.97	0.49	1.91
Hypersensitivity to PN	<0.001	3.08	1.52	6.27
Hypersensitivity to CF	0.07	0.60	0.35	1.04
Hypersensitivity to >1 BL antibiotic	0.5103	1.229	0.666	2.267
Hypersensitivity to other drugs	0.35	1.28	0.76	2.15
Time from BL administration to symptoms < 1 h	<0.001	0.15	0.07	0.30
Time from BL administration too symptoms ≥ 1–24 h	0.59	0.83	0.43	1.63
Time from BL administration to symptoms > 24 h	<0.001	6.64	2.80	15.74
Time from BL antibiotic administration to symptoms—unknown	0.01	2.13	1.23	3.70
>1 episode of hypersensitivity to BL antibiotic	0.01	2.13	1.22	3.73
HtD time ≥ 6 wks–6 months.	0.16	0.45	0.15	1.37
HtD time > 6 months–1 year	0.11	0.56	0.28	1.13
HtD time—several years	0.01	0.46	0.25	0.84
HtD time—10–20 years	0.16	1.75	0.80	3.82
HtD time—several decades	0.05	2.21	1.00	4.86
HtD time—unknown	0.01	2.26	1.19	4.30

BL—beta-lactam, CF—cephalosporins, HtD time—time from hypersensitivity episode to allergologic diagnosis, ns—non-significant, PAR—perennial allergic rhinitis, PN—penicillin, SAR—seasonal allergic rhinitis.

**Table 4:** Multivariate logistic regression model of the low-diagnostic-risk group presented as odds ratio (OR) with 95% confidence interval.

	Multivariate Model			
Variable	*p*	OR	95% CI	
HtD time—several years	<0.01	0.26	0.13	0.54
>1 episode of hypersensitivity to BL antibiotic	0.02	2.20	1.15	4.23
Time from BL antibiotic administration to symptoms < 1 h	<0.01	0.18	0.09	0.37
Time from BL antibiotic administration to onset of symptoms > 24 h	<0.01	4.71	1.80	12.32

BL—indiciates beta-lactam, HtD time—time from hypersensitivity episode to allergologic diagnosis.

## Data Availability

All obtained and analyzed data are included in this article. Further enquiries can be directed at the corresponding author.
